# Retrospective longitudinal study of osteoporosis in adults with recessive dystrophic epidermolysis bullosa

**DOI:** 10.1002/ccr3.1898

**Published:** 2018-11-12

**Authors:** Lynne D. Hubbard, Kattya Mayre‐Chilton

**Affiliations:** ^1^ Department of Nutrition and Dietetics Guy’s and St. Thomas’ NHS Foundation Trust London UK

**Keywords:** dermatology, epidermolysis bullosa, health maintenance, nutrition, osteoporosis

## Abstract

This retrospective study looks at bone mineral density of people with recessive dystrophic epidermolysis bullosa as assessed by dual‐energy x‐ray absorptiometry. Data were collected in 34 patients aged 16‐35 years. Statistical analysis showed immobility, low body mass index, and pubertal delay was associated with osteoporosis.

## 1 INTRODUCTION

Epidermolysis bullosa (EB) is a group of inherited disorders characterized by blistering of the skin and mucous membranes following mechanical trauma. Four main sub‐types are based on the location of blistering in the skin: EB simplex, junctional EB, dystrophic EB, and Kindler syndrome.^1^ This study looks at adults with recessive dystrophic epidermolysis bullosa (RDEB), which is caused by mutation of the COL7A1 gene that encodes the protein collagen VII, an essential component in anchoring fibrils at the dermal‐epidermal junction. Blistering is extensive and continual wound healing, loss of blood and plasma, and risk of infection mean nutritional requirements can be high. Nutritional intake can be compromised by blistering of the oro‐pharynx and esophagus. Recurrent blistering can result in scarring and esophageal strictures, requiring dilatation. In order to meet nutritional requirements, both supplementary drinks and enteral feed via a gastrostomy tube can be used to reduce the risk of malnutrition.^2^

Children with RDEB are at risk of developing osteoporosis—defined by the World Health Organization as a Bone Mineral Density (BMD) *Z* score of less than −2.5 standard deviations (SD) below normal and osteopenia as between −1 and −2.5 SDs below normal.^3^ At these levels, there is an increased likelihood of fracture. Risk factors for osteoporosis include reduced mobility, delayed puberty, inflammation, low body mass index, and inadequate intake of calcium and vitamin D.^4^ For people with EB, mobility may be reduced due to blistered feet, knee contractures, altered gait, muscle weakness, pain, and anemia. In a study of 39 children with EB aged 3‐17 years, Fewtrell^5^ found mobility to be a significant predictor of bone mineral content in children. Bruckner^6^ found low BMD of the lumbar spine correlated with low height and weight *Z* score, extensive blistering, immobility, and anemia. Fu^7^ examined changes in BMD in 17 children at an interval of 12‐25 months. Seven increased their BMD *Z* scores, 10 maintained or decreased their BMD, and the authors suggest that the rate of bone accrual was slower than in the general population. Cheung^8^ found that children with RDEB had vertebral fractures and low BMD *Z* scores that deteriorated with age. Pubertal delay was identified as a risk factor.

No studies have been conducted to investigate bone density in adults with EB and it is not known what happens during the period between puberty and age (approximately) 30 when peak bone mass (PBM) is attained in the healthy population.^9^ In this retrospective study, we looked at Dual‐Energy X‐ray Absorptiometry (DXA) scans to evaluate bone mass of adults with RDEB over time. We reported on known risk factors for osteoporosis, particularly mobility, puberty, body mass index (BMI), calcium intake, and vitamin D status as well as incidence of fracture. We recorded the reasons provided by patients for changes in their mobility and the types of activity in which they participated. In order to establish if bone health can improve during the period when PBM is accrued, we reported on the incidence of osteoporosis and changes in bone density from age 16‐35 years in adults with RDEB.

## 2 METHOD

All patients with RDEB attending an adult EB center in the UK were considered eligible for inclusion if they fulfilled the following criteria: age 16 years or older; had at least one DXA scan between 16 and 35 years of age; and were under the care of the EB multidisciplinary team.

The following data were collected from electronic patient records and from medical and dietetic notes, and recorded routinely:

1. gender and age (at 31 January 2015);
2. height centile attained,^10^ BMI;
3. DXA scan of the lumbar spine: the age of the patient at each scan was noted and assigned to one of four age categories (16‐20, 21‐25, 26‐30, and 31‐35 years) in order to compare patients at the same age;
4. mobility: a score was recorded on a four‐point scale, based on previous studies of bone health in EB^5^ (0, mainly wheelchair users; 1, walks between 10 and 25 meters; 2, walks more than 25 m; 3, walks normally), together with reasons for change in mobility and type of activity as reported by patients;
5. calcium and vitamin D: calcium intake was assessed using the diet history method, including information on dietary sources and supplementation (tube feed, oral nutrition support or dietary supplement) and the incidence of vitamin D deficiency based on blood levels;
6. bisphosphonate intake;
7. age of attainment of puberty;
8. fracture: site, cause, and age.

The EB sub‐type was obtained from medical notes and genotype classification by DNA and immunofluorescence as reported by the National Diagnostic EB Laboratory.

Descriptive statistics were used to identify the incidence of osteoporosis and osteopenia, changes in lumbar spine *Z* score, and the mean lumbar spine BMD *Z* score in each age category. Statistical analysis was used to explore the relationship between spine BMD *Z* score and pubertal delay, mobility and BMI.

The research study was submitted to the hospital's Research and Development Committee (assignment number 202614) and was given approval.

## 3 RESULTS

Thirty‐four patients met the inclusion criteria: 17 female and 17 male. The mean age in January 2015 was 31 years (range 17‐47). Twenty‐three patients (68%) had RDEB severe generalized (RDEB‐SG), seven (20%) had RDEB generalized intermediate (RDEB‐GI), and four (12%) had RDEB inversa (RDEB‐I): Table 1.

**Table 1:** Data on pubertal attainment, maximum height centile, lumbar spine *Z* score, and BMI according to EB sub‐type and age

	RDEB Sub‐type	Sex	16‐20 y			21‐25 y		26‐30 y		31‐35 y		Puberty (age in years)
			DXA	BMI	MHC	DXA	BMI	DXA	BMI	DXA	BMI	
1	SG	M	**−4.2**	10	25	—		**−2.6**	14	−2.4	14	Y (NK)
2	SG	F	**−**1.2	20	0.4‐2	−1.3	19	−1.9	19	—		Y (15)
3	SG	M	—	19	0.4‐2	**−3.9**	21	**−3.0**	24	—		Y(15)
4	SG	M	**−3.9**	14	<0.4	**−6.1**	14	—		—		N
5	SG	M	**−2.9**	16	50‐75	−1.5	16	—		—		Y (19)
6	SG	M	—	16	25‐50	−1.75	20	−1.7	23	—	23.5	Y (13)
7	SG	F	**−5.3**	15	<0.4	**−4.5**	15	—		—		N
8	SG	F	**−3.56**	18	0.4	**−4.0**	—	—	17.5	**−2.9**	18	Y (16)
9	SG	F	**−3.3**	16.5	25‐50	**−3.5**	16.5	—	1	—		Y (15)
10	SG	F	—	22.5	25	**−3.3**	20	**−3.5**	19.5	—		Y (13)
11	SG	M	—		0.4	−0.47	21	−1.39	19	—		Y (13)
12	SG	M	**−4.7**	16	<0.4	**−5.6**	12	—		—		N
13	SG	F	**−2.8**	19.5	2	−2.2		−2.2	18	—		Y (13)
14	SG	F	−0.45	15.5	50	−0.9	16	—		—		Y (13)
15	SG	F	−2.3	29	2	−2.3	17.5	−1.2	18	—	18	Y (15)
16	SG	F	—		2	—		**−2.6**	17.5	**−2.7**	17	Y (NK)
17	SG	F	—	17	9	**−2.6**	19.5	—	19.5	—	20	Y (13)
18	SG	F	—	13	50	**−3.9**	12	—	14			Y (17)
19	SG	M			<0.4					**−2.5**	19.6	N
20	SG	M			9					−1.86	18.5	Y (19)
21	SG	F			9‐25	—	15	−2.2	—			Y (NK)
22	SG	M	**−3.8**	22	<0.4							N
23	SG	F			<0.4			−2.0	26.5			Y (16)
24	GI	M	−2.2	15	91	−2.0	16	−1.3	15.5	—		Y (18)
25	GI	M	−0.66	—	50‐75	−0.7	18.5	−0.5	19	—	18.5	Y (14)
26	GI	M	−0.65	24	50	−0.9	24.5	—	24	—		Y (13)
27	GI	M	−0.65	20.5	2‐9	−2.0	22	−2.4	21			Y (NK)
28	GI	**F**	**−3.3**	17.5	99							Y (18)
29	GI	F			25					−0.27	20	Y (12)
30	GI	M			91‐98					−0.5	29.5	Y (15)
31	I	M	—		50	−1.2		−1.05	20	−0.7	22.5	Y (13)
32	I	M	—		25‐50	−1.9		−1.9	16.5	−1.4	25.5	Y (13)
33	I	F	−0.7	19.5	50							Y (17)
34	I	F			25			—	31	0.2	25	Y (NK)

DXA, numbers in bold indicate osteoporosis; MHC, maximum height centile attained; NK, not known.

Qualitative data on mobility were recorded from medical and dietetic notes on the type of weight‐bearing activity people with EB were undertaking, including walking, use of exercise bike, pilates, free weights, Nintendo Wifit, cycling, DJ‐ing, and dancing.^11^, ^12^ While activity was affected by physical restrictions associated with EB including knee contractures and blistered feet and legs, patients reported aspects of their social circumstances that induced them to increase or maintain their mobility including having children, working, and studying at university (Table 2).

**Table 2:** Social reasons reported for increased or maintenance of activity

Starting work	8
Starting university or college	6
Becoming a parent‐going to park and shopping	5
Learning to drive a car	1

Table 3 reports the mobility level by EB sub‐type at age 16‐20 years and at the end of the study (mean age 31 years), with mobility most affected in those with RDEB‐SG. For the purposes of statistical analysis, patients were divided into two groups, mainly wheelchair use and mainly walking. Statistical analysis (IBM SPSS Statistics version 22) of the mobility scores using the Fisher's exact test showed a statistical trend toward osteoporosis in those who were wheelchair bound compared with those who remained mobile (*P* = 0.069) in age group 21‐25 years.

**Table 3:** Mobility status at age 16‐20 y and end point of the study (n = 34)

	Activity at age 16‐20 by RDEB sub‐type	Activity at endpoint by RDEB sub‐type (mean age = 31 y)
Mainly wheelchair	RDEB‐SG = 4 (12%)	RDEB‐SG = 6 (18%)
10‐25 m	RDEB‐SG = 6 (18%)	RDEB‐SG = 4 (12%)
>25 m	RDEB‐SG = 9 (26%)	RDEB‐SG = 9 RDEB‐GI = 1 RDEB‐I = 1 Total = 11 (32%)
Walks normally	RDEB‐SG = 5 RDEB‐GI = 6 RDEB‐I = 4 Total = 15 (44%)	RDEB‐SG = 5 RDEB‐GI = 5 RDEB‐I = 3 Total = 13 (38%)

Puberty is the acquisition of secondary sexual characteristics associated with growth spurt and resulting in attainment of final adult height and reproductive function. It is known to be delayed in people with severe EB.^13^ In the UK, average age for menarche is 13 years in females and average age for achieving Tanner Stage 3 in males is 14. Information on age of menarche in females or age attaining Tanner Stage 3 in males was obtained from the notes or reported by the patient to the EB team. Results are reported in Table 1. Puberty was delayed in eight females and five males and not attained by one female and four males. By age 19 years, 29 (85%) patients had achieved puberty without requiring estrogen or testosterone treatment. The five patients (all with RDEB‐SG) who did not attain puberty had severe osteoporosis of the lumbar spine on DXA scans taken at age 16‐20 years, were immobile and were below the 0.4 centile for height by age 20. Statistical analysis using the chi‐squared test showed that those who did not attain puberty were significantly more likely to have osteoporosis than those who reached puberty (*P* = 0.029) in the 21‐25 years age group.

Twenty‐two patients had two or more DXA scans of the lumbar spine between the ages of 16 and 35 years. Ten patients (45%) had increased their BMD (seven of these with RDEB‐SG), seven (32%) had maintained their BMD, and five (23%) had decreased their BMD (Table 1). Improvement was greatest among those who attained puberty and improved or maintained mobility. Table 1 suggests that osteoporosis was higher among those with RDEB‐SG and even in those patients whose bone density improved after puberty they remained osteopenic. In patients with the milder RDEB‐I, all of whom achieved puberty and had normal mobility, osteopenia was still observed.

Vertebral lateral x‐ray was not performed in this EB center so no data exists on spinal fracture but three patients with RDEB‐SG had sustained other fractures; one of the neck of femur at age 30 and two of the ankle at age 22 and 30. All three had osteoporosis, were largely wheelchair bound and sustained falls.

The mean BMD *Z* score for the patients with RDEB‐SG was calculated as this was the largest group and facilitated comparison with other studies. The mean BMD *Z* score for the RDEB‐SG group was −3.14 at age 16‐20 (n = 12, SD = 1.31); −2.94 at age 21‐25 (n = 15, SD = 1.6); and −2.14 at age 26‐30 (n = 11, SD = 0.74). Although mean BMD *Z* score improved with age this was not statistically significant.

Data on vitamin D were too incomplete for statistical analysis. However, 16 (47%) patients on no supplements, fortified enteral nutrition or sip feeds were deficient (25(OH)D below 30 nmol/L) in vitamin D and were commenced on supplements.

Thirty‐two patients’ (94%) intake of calcium met the recommended nutrient intake (RNI). Two patients did not meet the RNI for calcium and their bone density had fallen. Five (15%) patients were taking bisphosphonate medication; of these, four patients had a second DXA scan: two showed improvement in bone density and two maintained bone density.

BMI below 18.5 kg/m^2^ has been identified as a risk factor for bone health. BMI improved with age in all RDEB sub‐types (Table 1). The chi‐squared test was used to compare BMI below 18.5 kg/m^2^ with risk of osteoporosis and found to be significant (*P* = 0.029) in the age group 16‐20.

Height was measured in 34 patients (Table 1) and the maximum centile attained as follows: six (18.4%) <0.4th centile; 11 (32%) between 0.4‐<25th centile; 14 (41%) 25‐75th centile; three ≥91st centile. Small stature was higher in those with RDEB‐SG. Twenty‐one patients had their height measured at aged 18 and again between aged 21 and 25: in nine (30%) height had increased, suggesting growth was occurring later than in the non‐EB population possibly due to pubertal delay.

## 4 DISCUSSION

Patients with severe types of EB are at high risk of osteoporosis of the lumbar spine compared with the non‐EB population of the same age and this can result in fracture. Even people with RDEB‐ I, a more localized form of RDEB in which mobility remains normal, show some risk of osteopenia.

Our results suggest that when puberty is attained, it is possible for adults with RDEB to improve bone density of the lumbar spine. If this is not achieved, however, optimization of mobility, BMI and nutrition seem to have little or no effect. Martinez^13^ reviewed the possible causes of non‐attainment or delay of puberty in EB. She identified the impact of malnutrition on the hormone system regulating puberty and growth (leptin, insulin, thyroid hormones, cortisol, growth hormone, and the hypothalamo‐pituitary axis) and the chronic increase in pro‐inflammatory cytokines (interleukin‐6 and tumor necrosis factor). Cheung^8^ found bone density to be low (*Z* score −3.9 ± 1.5) in adolescents with RDEB‐SG (mean age 15 years, n = 20). In her study, only 16% of participants attained puberty: Cheung suggested that failure to attain puberty was likely to be an important factor in low BMD. In our study, 79% of patients with RDEB‐SG reached puberty without hormone therapy, although this was delayed in 32% of participants. The mean BMD *Z* score at age 16‐20 was −3.14 ± 1.31 (n = 12); at age 21‐25 it was −2.94 ± 1.6 (n = 15); and at age 26‐30 it was −2.14 ± 0.74 (n = 11). These figures suggest that attainment of puberty can lead to an improvement in bone density in the lumbar spine. The five patients who did not attain puberty were referred to the endocrinologist for treatment: In one patient before transfer at age 17 years and in four after the age of 20 years. Although discussions regarding delayed puberty commenced before this at the pediatric EB center,patients often were reluctant to begin hormonal intervention.

Weight‐bearing activity stimulates both production of osteoblasts and bone mineralization. Studies of children with EB^5^ have found mobility to be a significant factor in improving BMD: the results of our study suggest this is also important in adults with RDEB. Mobility can be reduced in EB due to physical problems including blistered feet, knee contractures, pain, and anemia.^14^ Our study also identifies the importance of social issues in motivating patients to be mobile: some adults reported that they remained mobile in order to work, to attend university, or to care for their children. When people are excluded from these activities and become socially isolated, their motivation to remain mobile can decrease. Some adults in our study were motivated to try to maintain muscle strength required for mobility and participated in a wide range of activities ranging from using weights to dance and pilates.^11^, ^12^ We suggest that maintaining and improving mobility in RDEB should be approached holistically. Physiotherapists can assess and optimize gait, contracture and muscle strength,^15^ while podiatrists can use gait assessment to advise on footwear that reduces blistering to the feet.^16^, ^17^ Mobility not only increases bone accrual but also contributes to muscle strength, balance, and coordination, reducing the risk of falls and fractures.^18^

BMI below 18.5 kg/m^2^ was also found to be a significant factor associated with osteoporosis. This is the case in the population generally and is possibly a reflection of malnutrition, low body mass, and reduced muscle strength.

The majority of patients in this study met the RNI for calcium as dairy products are soft in texture and have a neutral pH so people with EB find them easy to swallow, with a low risk of trauma to the esophagus. However, the bone density of the two patients who did not achieve the RNI for calcium fell over time. Because patients with EB avoid exposure to the sun and milk is not fortified in the UK, vitamin D levels were often deficient unless patients were on supplements. The role of calcium and vitamin D needs further evaluation in a prospective study.

Bisphosphonates are rarely prescribed for treatment of osteoporosis at the EB center because of concerns about osteonecrosis of the jaw.^19^ People with RDEB are at high risk of dental caries due to difficulty maintaining good oral health, as a result of microstomia and blistering of the oral cavity: many require major dental repair and even dental clearance later in life. However Martinez^20^ reports on the successful use of IV Pamidronate for treatment of vertebral fractures in children with RDEB and the use of Risedronate in bone pain. Further discussion on the use of bisphosphonates in adults with RDEB is needed. Alternatives to bisphosphonates are available: guidelines developed by the UK's National Institute for Clinical Excellence (NICE) guidelines recommend strontium ranelate and teriparatide as alternative treatments in the prevention of osteoporotic fragility fractures in post‐menopausal women.^21^ One case report has been published on the use of teriparatide as a treatment for osteoporosis in EB.^22^

Risk of vertebral compression fracture and of scoliosis has also been identified in children with RDEB.^20^ Cheung^8^ found that in children aged seven to eight years (n = 22), 27% had vertebral compression fractures and 5% had scoliosis, while among children aged 15‐18 years (n = 15) 38% had vertebral compression fractures and 33% had scoliosis. These problems were identified using plain lateral x‐rays of the thoracic and lumbar spine. X‐rays to identify vertebral fractures are not currently performed in adults with EB at our center and it is well recognized that these can go unreported in the population at large. They can lead to scoliosis resulting in pain and postural change, with the effect of limiting activity.^23^ DXA scans will not identify spinal fracture and we recommend that annual lateral x‐rays of the thoracic and lumbar spine are needed to identify spinal fracture.During the study period, however, three patients sustained fractures of the hip or ankle due to falls. Subsequently, a further patient sustained a complex fracture of the femur. Due to the risk of osteomyelitis, surgical intervention was not carried out and the patient died.

This study was limited by the small sample size; its retrospective nature also meant data was sometimes incomplete. We plan to continue collecting data prospectively, increasing the size of the sample size for future statistical analysis.

DXA scans every 2 years to assess changes in bone density and lateral x‐rays annually of the thoracic and lumbar spine to identify vertebral fracture should be part of a comprehensive skeletal health assessment for RDEB patients with the aim of reducing fractures and their impact on morbidity and mortality. This study indicates that if patients with RDEB attain puberty, maintain mobility and optimize nutrition, it is possible to improve BMD while PBM is being accrued. The study also identifies the need to instigate monitoring of patients for vertebral fractures using lateral spine x‐ray and to review optimal medication, including bisphosphonates, for treatment of osteoporosis.

Future research needs to look at the benefits of an intervention program beginning at adolescence that aims to optimize BMD and to increase muscle strength, balance, and coordination. This could include education on bone health and nutrition; individualized activity programmes; and optimization of gait and footwear. The combined outcome may reduce the risk of falls and associated fracture risk and help maintain mobility, which remains central to independence and quality of life.^24^

## CONFLICT OF INTEREST

None declared.

## AUTHOR CONTRIBUTION

LH: conceived of the research, analyzed the data, and wrote the paper. KM‐C: assisted in data analysis, discussed the findings, and checked the paper.
